# Adherence to the Mediterranean diet among adults in Mediterranean countries: a systematic literature review

**DOI:** 10.1007/s00394-022-02885-0

**Published:** 2022-04-22

**Authors:** Cecile A. Obeid, Jessica S. Gubbels, Doris Jaalouk, Stef P. J. Kremers, Anke Oenema

**Affiliations:** 1 Department of Health Promotion, NUTRIM School of Nutrition and Translational Research in Metabolism, Maastricht University Medical Centre, PO Box 616, 6200 MD Maastricht, The Netherlands; 2 Faculty of Nursing and Health Sciences, Notre Dame University, Zouk Mosbeh, PO Box 72, Zouk Mikael, Lebanon; 3 College of Arts and Sciences, American University of Iraq Baghdad (AUIB), Airport Road, Baghdad, Iraq

**Keywords:** Mediterranean diet, Adherence, Mediterranean countries, Adults

## Abstract

**Background and aim:**

While the Mediterranean diet (MD) is promoted in non-Mediterranean countries, inhabitants of Mediterranean countries seem to be shifting away from this healthy diet. The aim of this study is to provide an overview of MD adherence in the general adult population of Mediterranean countries.

**Methods:**

A systematic review was conducted following the PRISMA 2020 (Preferred Reporting Items for Systematic Review and Meta-Analysis) guidelines and registered in the Prospero database (CRD42020189337). Literature was searched in PubMed, Web of Science and PsycINFO databases for studies published from 2010 up to and including 2021. The following inclusion criteria were used: age 18 years and older, sample size > 1000 participants, and using a validated MD adherence score. Studies that only included participants with nutrition-related or other severe chronic disorders, as well as studies that only included specific subpopulations (e.g., pregnant women), were excluded in order to focus on the general adult population. A quality analysis of the included studies was done using the NCCMT scale.

**Results:**

A total of 50 studies were included. The number of participants in the included studies ranged between 1013 and 94,113. Most of the included studies pertained to the European Mediterranean countries, with fewer studies from the Middle Eastern and North African Mediterranean countries. The vast majority of the included studies reported low or moderate MD adherence, both based on the mean adherence as well as the low or moderate adherence category often being the most prevalent. There were no clear differences noted between sex and age groups. The quality assessment generally showed weak or moderate scores.

**Conclusions:**

Mediterranean populations have been showing moderate adherence to MD in the past 10 years, indicating room for improving adherence to the MD in countries of its origin.

**Supplementary Information:**

The online version contains supplementary material available at 10.1007/s00394-022-02885-0.

## Introduction

There is growing evidence that the Mediterranean diet (MD) is associated with better health outcomes through the prevention of a variety of chronic diseases. According to various systematic reviews and meta-analyses, the MD has been associated with a reduced risk for cardiovascular diseases [1]. The MD has also been found to be associated with better control of glycemia, blood pressure levels, lipid panel, and inflammatory markers, resulting in improved control of cardiovascular risk factors and better management of diabetes type II [2, 3]. Moreover, the MD was found to protect against fragility of the elderly [4, 5], Alzheimer’s disease, dementia [6], and depression in different age groups [7, 8]. A recently published systematic review further reported that the MD is associated with a lower risk of various types of cancer, as well as a reduced risk of mortality from cancer among the general population and cancer survivors specifically [9, 10].

Originally, the MD was the dietary pattern described as the one followed by populations of olive tree-growing areas around the Mediterranean basin (Albania, Algeria, Bosnia, Croatia, Cyprus, Egypt, France, Gibraltar, Greece, Israel, Italy, Lebanon, Libya, Morocco, Malta, Monaco, Montenegro, Palestinian territory, Slovenia, Spain, Syria, Turkey, and Tunisia) [11]. The MD is defined by generous consumption of whole grains, fruits, vegetables, nuts, seeds, and legumes, olives and olive oil as the main source of fat intake, regular but moderate intake of dairy products (milk, yoghurt and cheese), moderate consumption of fish, and very limited intake of processed food, meat and meat products, in addition to moderate wine drinking (with meals) [12, 13]. This dietary pattern is characterized by high levels of unsaturated fatty acids type ϖ3, polyphenols, vitamin D and B group vitamins, in addition to complex carbohydrates that play a favorable role in health outcomes [6].

Since the benefits of the MD are increasingly being recognized, many studies have examined adherence to it across the world. Inhabitants of countries that traditionally do not have a MD are increasingly adopting this dietary pattern due to its healthy virtues. A study among the elderly across the US found a moderate adherence to the MD [14]. In addition, a study of the Australian population found relatively good adherence among Australian females [15]. However, although a systematic overview of evidence is lacking as yet, there seems to be a trend of decline of adherence to the MD in many Mediterranean countries [13, 16–19]. For instance, Veronese et al. noted a significant decrease in adherence to MD in Italy between the years 1985–1986 and 2005–2006, which was more prominent among younger than older participants, and was mainly caused by a reduction in olive oil consumption [20]. The reasons behind this decline are suspected to be diverse. Bonnacio et al. believe that socio-economic factors play a major role in the shift from the MD toward more Western diets and increased use of convenience foods [21]. Naja et al. found that food insecurity affects adherence to MD negatively among Lebanese adolescents [22]. The impact of age on adherence to the MD is unclear, as some studies reported a decrease of adherence with age due to loss of interest in food, chewing difficulties, financial hardships after retirement, or dependency in food preparation, while others found an increase in MD adherence with age, possibly due to the increase in nutrition-related disorders with age, requiring dietary changes which usually follow the Mediterranean dietary pattern [12]. Sex differences in adherence to the MD have also been extensively researched and results are inconclusive: while some found better adherence in women, mainly due to lower red meat consumption [23], others did not find a difference between men and women [24].

The declining trends in adherence to the MD in Mediterranean countries may indicate that it is important to promote MD adherence, even in countries where it originated due to its numerous health virtues in preventing several diseases. A starting point for MD adherence promotion is to identify the level of adherence in the general population, as well as in specific subgroups (age and sex groups), which can provide valuable input for national nutrition policies of Mediterranean countries. The aim of this study is to provide an overview of MD adherence in the general adult population living in a Mediterranean country (i.e., Albania, Algeria, Bosnia, Croatia, Cyprus, Egypt, France, Gibraltar, Greece, Israel, Italy, Lebanon, Libya, Morocco, Malta, Monaco, Montenegro, Palestinian territory, Slovenia, Spain, Syria, Turkey, and Tunisia). In addition, this study will examine differences in adherence in various age and sex groups.

## Methodology

A systematic review of studies reporting on adherence to the MD among adults from Mediterranean countries was conducted as per PRISMA 2020 guidelines [25]. The review protocol was registered in the Prospero database under the registration number: CRD42020189337.

### Selection criteria for studies

The inclusion criteria were the following: studies conducted among adults (mean age above 18 years in the studied sample), living in a Mediterranean country (i.e., Albania, Algeria, Bosnia, Croatia, Cyprus, Egypt, France, Gibraltar, Greece, Israel, Italy, Lebanon, Libya, Morocco, Malta, Monaco, Montenegro, Palestinian territory, Slovenia, Spain, Syria, Turkey, and Tunisia) and using a validated dietary assessment and scoring tool to quantify adherence to the MD (e.g., the Greek Mediterranean Index (MedDietScore) [26] or the Mediterranean Diet Scale (MDS) [12]). We included the study if it reported either a mean or a median adherence score or a distribution of adherence categories (e.g., low, moderate, high) in the general population and/or in subgroups for age and/or sex. Studies that *solely* included populations with chronic illnesses, co-morbidities, or a high risk of nutrition-related disorders (e.g., inflammatory bowel diseases, cardiovascular diseases, diabetes, kidney diseases, or wasting diseases such as cancer and HIV), or with a condition that affects the ability to independently choose food intake (e.g., documented dementia, Alzheimer’s disease or psychological disorders such as schizophrenia) were excluded. We also excluded studies among specific subpopulations such as pregnant women, centenarians or athletes, to focus on the general population. In addition, we excluded studies with a sample size of less than 1000 participants, aiming for representativeness of the general population as well as studies assessing MD adherence during the COVID 19 pandemic lockdown as it does not reflect the normal lifestyle of the general population. When there were multiple studies reporting on the same cohort or sample, we retained the study that had the least exclusion criteria (assumed to be the most representative of the broader general population), and that reported on either mean/median adherence score and/or distribution of adherence categories in the general sample. If means/medians and distribution were reported in separate studies, we included two studies that provided complementary data regarding the same cohort or sample. In addition, only observational studies (prospective cohorts and cross-sectional studies) published during the past 10 years (2010 or later) and in the English language were retained.

### Literature search

To perform a comprehensive search of the literature, three databases were searched until January 2022: PubMed, PsycINFO and Web of Science. The search strategy was formed by a combination of controlled descriptors (indexers in each database) and keywords, according to the indication offered in each electronic database. The final search strategy was the result of various iterations to arrive at the most optimal search strategy.

We used a filter to retain studies published in the English language between 1st January 2010 and 24th January 2022. The search strategies for all the databases can be found in appendix S4.

**Fig. 1:**
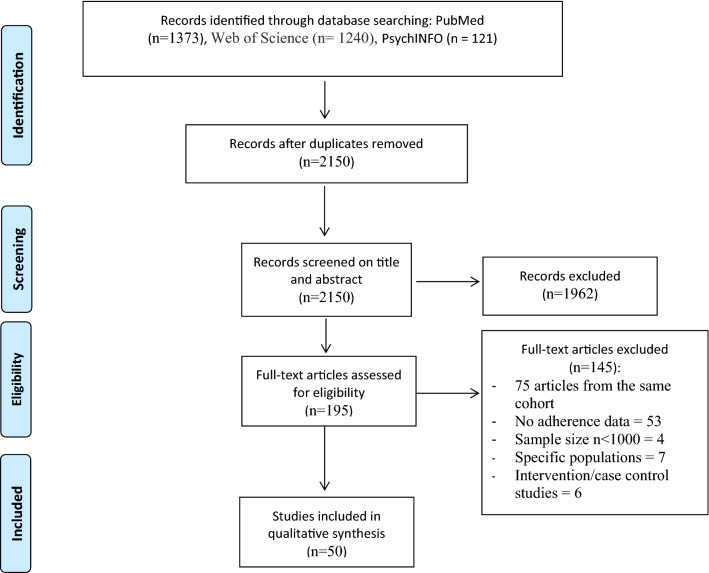
Flow chart result of the search strategy

After identifying the records, the selection process was done using Rayyan Qatar Computing Research Institute (QCRI) software. After removing duplicates (performed by CO), articles were screened against the inclusion and exclusion criteria first on the title and then on the abstract (CO). Excluded articles were then confirmed by a second author (DJ), discrepancies were resolved by discussion and confirmed with the other authors (JG and AO). Then full texts were screened for final selection by CO and DJ. The final number of included articles was 50 (Fig. 1).

### Data extraction and quality assessment

Data were abstracted for each included article and tabulated, in an Excel file, by two authors (CO and DJ). Disagreements were resolved through consensus with a third person (JG/AO). The following data were extracted from the articles: name of first author and year of publication, study design, date of data collection, general population’s characteristics (nationality, sample size, age, and sex, education level, socio-economic status, marital status, urban/rural living and BMI), the dietary intake assessment tool and the score used to assess MD adherence. In addition, the mean of MD adherence and/or distribution across categories in the general population and, if reported, the distribution or mean of MD adherence per age and sex subgroups were extracted. If MD adherence was reported at different points in time, the mean MD adherence score was reported for all time points [26, 27] (Table 2).

Quality assessment was conducted using a selection of items from the National Collaborating Center for Methods and Tools (NCCMT) scale for assessment of quantitative studies [28]. The NCCMT tool incorporates in total seven aspects of study quality. Three aspects were selected which were relevant for the type of studies included in the present review (i.e., focusing on providing a cross-sectional description of adherence to the MD). This included an assessment of the aspects ‘selection bias’, ‘data collection methods’, and ‘withdrawals and drop-out’. Each of these aspects were assessed using two items, based on which a quality score in terms of low, moderate or high was assigned for each aspect. We used the NCCMT rating system with a slight modification for calculating an overall quality score as follows: strong (only strong ratings), moderate (one weak rating or no weak ratings but mostly moderate ratings) and weak (two or more weak ratings). The overall rating was added if all three aspects were scored, otherwise the study’s overall score would not be applicable. The quality assessment was performed by JG and AO. Ten percent of the papers (five studies) were assessed independently by both reviewers to ensure inter-rater reliability. Disagreements were discussed until consensus was reached, after which the remaining studies were assessed by one or both authors (JG and/or AO).

### Data analysis

To be able to compare the mean MD adherence score reported by the included studies, the mean adherence score was translated into a qualitative interpretation of the findings in terms of high, moderate, and low adherence to the MD. This labeling of the means as either high, moderate or low was based on the common classifications used for the different scoring systems in the included articles, as shown in Table 1. If the paper reported according to multiple scoring systems, we reported on the scores mentioned in Table 1, which are in general the most frequently used scores.

**Table 1:** Overview of the Mediterranean diet scores systems that were used in the included studies

Name of Scoring system	Range of MD adherence score for classification in categories		
	Low	Moderate	High
Mediterranean diet scale (MDS) by Trichoupoulo et al. (2003) [12]	0–2.9	3–5.9	6–9
Italian Mediterranean Index (IMI) by Agnoli et al. (2013) [29]	0–2.9	3–5.9	6–9
rMed by Buckland et al. (2010) [30]	0–6.9	7–10.9	≥ 11
MedDietScore by Panagiotakos et al. (2006) [13]	0–32.9	33–36.9	37–55
MEDAS by Schroder et al. et al. (2011) [31]	0–6.9	7–10.9	11–14
Lebanese Mediterranean Index (LMD) by Naja et al. (2015) [32]	9–14.9	15–20.9	21–27
Mediterranean Diet Scoring System (MDSS) by Monteagudo et al. (2015) [33]	0–9.9	10–13.9	≥ 14
Medi-Lite score by Sofi et al. (2017) [34]	0–10.9	11–14.9	≥ 15

## Results

### Study selection

The search of the 3 databases yielded a total of 2734 studies (Fig. 1). After removing duplicates (*n* = 584), 2150 unique articles remained. Subsequent to the screening of articles’ titles and abstracts, 1962 were excluded. The number of retained articles for full text screening was 195. Among these 195 articles, 83 articles were related to the same cohorts and were distributed as follows: ATTICA (18 studies), EPIC (15), MOLI-SANI (9), SUN (17), Three-city population (3), HELIAD (4), SU.VI.MAX (3), MEAL (7), MEDIS (4), Seniors-ENRICA (3). In total, we included 14 of these articles, as explained in the methodology section. Other reasons to exclude articles were the lack of MD adherence data, being focused on specific populations (e.g., athletes or health majors’ students), studies assessing MD adherence during the COVID 19 pandemic lockdown and studies that did not fulfill the criteria of age (< 18 years old) and sample size (< 1000 participants). The final number of included studies was *n* = 50. The result of the selection procedure is summarized in Fig. 1 [35].

### Characteristics of included studies

Included studies conducted in Spain (*n* = 17) formed the largest group, followed by nine in France, nine in Italy, six from Greece, two from Lebanon, one each from Israel, Croatia, Malta, Cyprus and three papers reporting data from various Mediterranean countries (Table 2). In total, 27 studies had a cross-sectional design, and 23 papers were derived from prospective cohorts. The age of the included populations ranged between 18 and 95, with 10 studies including older populations aged > 60 years only. The vast majority of the studies were performed on a community-based sample and presented data on both males and females. Three studies [36–38] included females only. The number of participants in the included studies ranged between 1013 and 94,113. Twenty three papers reported results of MD adherence by sex group and eight studies reported results of adherence by age group. Results on MD adherence (as mean, median and/or distribution of categories) from the included papers are shown in Table 3. The mean MD adherence score for the total population was reported in 35 papers, 5 papers reported the median MD adherence score, and the remaining papers reported the distribution of MD adherence by categories. The MDS by Trichoupoulo et al. (0–9 or 0–8) was used in 15 papers [16, 32, 36, 38–49], whereas the rMed score (0–17 or 0–18) was used in nine papers [30, 32, 50–56] and the MedDietScore by Panagiotakos et al. score (0–55) were used in ten papers [8, 20, 26, 32, 57–62]. Other scores were used in fewer studies, such as the Italian Mediterranean Index (IMI) or the Lebanese Mediterranean Index (LMD) [12, 26, 29, 30, 32].

**Table 2:** Overview of study design, sample size and sample characteristics of the included studies clustered by country

	References	Study design (name of cohort)	Sample size	Sample characteristics (sociodemographic)*
**France**				
1	Féart et al. (2011) [39]	PC (Three-City 3C)	1410	Age ≥ 65; mean age 75.9 (67.7 – 94.9) 37.3% males
2	Féart et al. (2012) [16]	PC (Three-City 3C)	1595	Age ≥ 65; mean age: 76.1 (67.7–94.9) 38.1% males
3	Kesse-Guyot et al. (2013) [40]	PC (SU.VI.MAX.2)	3083	Mean age: 52.0 ± 4.6 53.7% males
4	Barré et al. (2017) [36]	PC (French E3N Cohort)	64,052	Mean age: 52.7 100% females 36% high education level Mean BMI: 22.8
5	Trebuchet et al. (2019) [50]	PC (NutriNet-Santé)	94,113	Age > 18; mean age: 43.9 ± 14.6 21% males post-secondary education 64.3% Mean BMI: 23.84 ± 4.57
6	Adjibade et al. (2018) [51]	PC (SU.VI.MAX)	3523	Mean age: 49.5 ± 6.2 42.3% males
7	Lavalette et al. (2018) [52]	PC (NutriNet-Santé)	41,543	Age ≥ 18; mean age: 54.6 ± 8.7 26.5% males 73.7% post-secondary education Mean BMI: 24.5 ± 4.5
8	Lelong et al. (2016) [41]	PC (NutriNet-Santé)	11,302	Age ≥ 18; mean age: 51.7 ± 13.5 24.6% males 68.2% had university or equivalent Mean BMI: 23.8 ± 4.0
9	Lassale et al. (2012) [42]	PC (SU.VI.MAX)	3151	Age range: 45–60; mean age: 52.3 ± 4.6 53.3% males 40.8% university graduates Mean BMI: 24.4 (3.4)
10	Buckland et al. (2010) [30]	PC (EPIC)	180,718	Age range: 35–70
**Greece**				
11	Koustonida et al. (2021) [63]	PC (EHS)	1273	Age range: 21–77; mean age: 47.82 ± 11 40.55% males 67% High education Mean BMI: 26.41 (4.68)
12	Mantzorou et al. (2021) [8]	CS	2092	Age > 65; mean age: 74.97 ± 8.41 48% males
13	Mamalaki et al. (2020) [57]	CS (HELIAD)	1993	Age ≥ 65; mean age: 73 ± 6 41% males Mean BMI: 28.9 ± 4.7
14	Mamalaki et al. (2018) [58]	CS (HELIAD)	1639	Age ≥ 65; mean age: 72.7 ± 5.7 41% males Mean BMI: 28.9 ± 4.7
15	Maraki et al. (2019) [59]	CS (HELIAD)	1731	Age ≥ 65; mean age: 73 ± 6 41% males Mean BMI: 28.9 ± 4.7
16	Panagiotakos et al. (2015) [26]	PC (ATTICA)	3042	Age range: 18–89; mean age: 46 ± 14 49.8% males 78% urban dwellers Mean BMI: 26 ± 5
**Spain**				
17	Zazpe I et al. (2021) [49]	PC (The SUN)	5515	Age > 20; Mean age: 36.3 years (10.7) 41% males
18	Gutiérrez-Carrasquilla et al. (2019) [64]	CS (ILERVAS)	3020	Age range: 45–70
19	Navarrete-Muñoz et al. (2018) [53]	PC (DiSA-UMH)	1026	Age range: 17–35 28% males Health sciences university students
20	Galilea-Zabalza et al. (2018) [54]	PC (REDIMED-PLUS)	6430	Age range: 55–75
21	Cornejo del Rio et al. (2017) [65]	CS (SPREDIA-2)	1586	Mean age: 61.5 (6) 43% males 32% university education
22	Ferreira-Pêgo et al. (2017) [66]	CS	1262	Age ≥ 18 50% males
23	Domınguez et al. (2013) [43]	PC (The SUN)	20,155	Mean age: 38.4 39.5% males
24	Mateo-Gallego et al. (2017) [44]	CS (The Aragon Workers' Health)	2588	Mean age: 51.3 ± 3.89 94.9% men
25	Olza et al. (2019) [44]	CS (ANIBES)	2286	Age range: 19–75 51% males
26	Sayon-Orea et al. (2015) [37]	CS	8954	Mean age: 54.3 ± 6.6 100% females population-based peri-/post-menopausal women 60.0% had high school/university education Mean BMI: 25.4 (± 4.5)
27	León-Muñoz et al. (2012) [18]	CS (ENRICA)	11,742	Age ≥ 18 49.5% males Representative of the population aged ≥ 18 28.2% had university education 39.2% had normal BMI
28	Campanini et al. (2017) [67]	PC (Seniors-ENRICA)	1596	Age ≥ 60 years Population-based sample
29	Alemán et al. (2016) [24]	CS (DIMERICA)	1732	Age > 20; median age: 51 47% males Healthy volunteers 44.2% had university degree Mean BMI: 25.6 (22.7–29.2)
30	León-Muñoz et al. (2014) [46]	PC (Seniors-ENRICA)	1815	Age ≥ 60 years Population-based adult sample
31	Rodríguez-Mireles et al. (2018) [27]	CS	4160 (2009); 4143 (2015)	Age > 16; mean age: 47.6 ± 17.2; 50.8 ± 16.8 (2009; 2015, respectively) 41%—43% males (2009; 2015, respectively); 50%- 54% secondary education (2009; 2015, respectively) Mean BMI: 2009: 26.2 (± 4.83), 2015: 26.21 (± 4.71)
32	Moreno-Agostino et al. (2019) [68]	CS (COURAGE in Europe)	2397	Age range: 21–101; mean age: 61.9 ± 15.2 46% males 86% urban dwellers 43% retirees 31% employed 41.23% were overweight
33	Garcıa-Arenzana et al. (2012) [38]	CS (DDM-Spain)	3564	Age range: 45–68; mean age: 56.2 ± 5.5 100% females 28.9% > secondary education 41.7% had normal BMI
**Italy**				
34	Dinu M et al. (2020) [69]	CS (Medi-Lite)	1820	Age > 18; 46.3% aged 18–30 y 39.6% males 52.1% unmarried/single 48.4% university degree
35	Ruggiero et al. (2019) [17]	PC (INHES)	7430	Age > 20; 65% aged 35–64 y 46% males 42% upper secondary education; 17% post-secondary 86% urban dwellers 32% retirees 49.8% had normal BMI
36	Barrea et al. (2017) [70]	CS	1013	Age range: 8–58; mean age: 37 (18–58) 46% males Mean BMI: 33.5 (19.5–57.9)
37	Limongi et al. (2017) [71]	PC (ILSA)	4232	Age range: 65–84
38	Zappala et al. (2019) [55]	CS (MEAL)	1936	Age ≥ 18 Urban dwellers
39	Marventano et al. (2018) [56]	CS (MEAL)	1937	Age ≥ 18 Urban dwellers
40	Bertoli et al. (2015) [72]	CS (ICANS)	4388	Age range: 18–80; median age: 46 26.8% males Mean BMI: 27.9 (25.0–31.0)
41	Bonaccio et al. (2012) [47]	PC (Moli-sani)	13,262	Age ≥ 35 years; mean age: 53.3 ± 10.6 49.7% males
42	Veronese et al. (2020) [20]	CS (MICOL)	2451 (1985–1989) 2375 (2005–2006)	Age range: 30–69 55%-60% males
**Other Mediterranean countries (Malta, Cyprus, Croatia, Lebanon, Israel, and MD islands)**				
43	Cuschieri S et al. (2021) [73]	CS	3947	Age range: 18–70; mean age 44.8 ± 15.1 50.6% males
44	Kyprianidou et al. (2020) [62]	CS	1140	Age > 18; mean age: 41 ± 17 43.6% males 54% married, 64% higher education 40% private employees Mean BMI: 25 ± 5 kg/m^2^
45	Quarta S et al. (2021) [74]	CS (MeDiWeb)	2163	Age > 18; 32.8% males Mean BMI: 24.6 (± 4.5)
46	Foscolou et al. (2018) [60]	PC (MEDIS)	3131	Age > 65
47	Cherfan et al. (2018) [61]	CS	2014	Age > 20; mean age: 41.3 ± 17.0 48.5% males 46% university education 52.55 urban dwellers 62% employed Mean BMI: 26.8 (± 4.9)
48	Kolčić et al. (2016) [75]	CS (10,001 Dalmatians)	2768	Age ≥ 18; median age: 55.0–58.0 36.6–39.7% males
49	Naja et al. (2015) [32]	CS	2048	Age range: 20–55; mean age: 34.7 ± 9.9 45.1% males Nationally representative adult sample 34.1% had university and higher education
50	Zbeida et al. (2014) [48]	PC (MABAT ZAHAV)	1786	Age ≥ 65 50% males Community-dwelling representative sample

*PC* prospective cohort, *CS* cross-sectional

*We reported the data (on the total general population) that was presented in the studies.

**Table 3:** Adherence scores and distribution of population by categories of MD adherence in the included studies

	Author(s)	Sample size	Mean score (± SD)	Classification of mean	Distribution of population (%) by categories of MD adherence^(k)^				
					Low		Moderate		High
	**France**								
1	Feart C et al. (2011)^a^ [39]	1410	4.4 (1.7)	Moderate	30.0		**43.6**		26.4
2	Feart C et al. (2012)^a^ [16]	1595	4.36 (1.67)	Moderate	30.8		**43.5**		25.7
3	Kesse-Guyot E et al. (2013)^a^ [40]	3083	4.6 (1.6)	Moderate	27.0		**45.6**		27.3
4	Barré A et al. (2017)^a^ [36]	64,052	NR		28.4		**44.1**		27.4
5	Trebuchet A et al. (2019)^b^ [50]	94,113	9.61 (2.77)	Moderate	23.3		**50.9**		25.7
6	Adjibade M et al. (2018)^b^ [51]	3523	NR		30.4		**41.0**		28.5
7	Lavalette C et al. (2018)^b^ [52]	41,543	8.4 (2.3)	Moderate	NR				
8	Lelong et al. (2016)^a^ [41]	11,302	4.3 (1.6)	Moderate	NR				
9	Lassale et al. (2012)^a,b^ [42]	3151	MDS = 4.5 (1.6) rMED = 9 (2.8)	Moderate Moderate	NR				
10	Buckland et al. (2010)^b^ [30]	68,892	9.5 (2.6)	Moderate	NR				
	**Greece**								
11	Koustonida et al. (2021)^d^ [63]	1201	7.25 (1.74)	Moderate	**54.1**		NR		45.9
12	Mantzorou et al. (2021)^c^ [8]	2092	28 (11–42)^j^	Low	**52.1**		24.9		23
13	Mamalaki E et al. (2020)^c^ [57]	1993	33·3 (4·6)	Moderate	32.9		**33.5**		30.5
14	Mamalaki E et al. (2018)^c^ [58]	1639	33.4 (4.5)	Moderate	32.2		**34.3**		33.5
15	Maraki M et al. (2019)^c^ [59]	1731	33.2 (4.6)	Moderate	26.7		**45.5**		24.5
16	Panagiotakos D et al. (2015)^c^ [26]	2001–02: 3042 2006: 2101 2011–12: 2583	2001–02:26 (7) 2006:25 (7) 2011–12:25 (7)	2001–02: Low 2006: Low 2011–15: Low	NR				
10	Buckland et al. (2010)^b^ [30]	25,984	12.9 (1.9)	High	NR				
	**Spain**								
17	Zazpe I et al. (2021)^a,d^ [49]	5515	Baseline (1999): MEDAS: 6.2 (1.7) MDS: 4.3 (1.8) 10 years follow up: MEDAS: 7.2 (1.7) MDS: 4.4 (1.7)	Baseline (1999): MEDAS: Low MDS: Moderate 10 years follow up: MEDAS: Moderate MDS: Moderate	35.8		**54.2**		10
18	Gutiérrez-Carrasquilla L et al. (2019)^d^ [64]	3020	NR		12.4		**80.1**		7.4
19	Navarrete-Muñoz E et al. (2018)^b^ [53]	1026	NR		25.3		**50.1**		24.6
20	Galilea-Zabalza I et al. (2018)^b^ [54]	6430	NR		24.4		52.5		23.1
21	Cornejo del Rio V et al. (2017)^d^ [65]	1586	8.6 (2.1)	Moderate	NR		NR		18.7
22	Ferreira-Pêgo C et al. (2017)^d^ [66]	1262	NR		24.3		**41.0**		34.7
23	Domınguez et al. (2013)^a^ [43]	20,155	NR		22.0		**62.9**		15.1
24	Mateo-Gallego R et al. (2017)^a^ [44]	2566	NR		16.9		**60.4**		22.7
25	Olza J et al. (2019)^a^ [45]	2286	NR		44.8		NR		**55.2**
26	Sayon-Orea et al. (2015)^d^ [37]	8954	7.6 (2.0)	Moderate	**47.6**		35.7		16.7
27	León-Muñoz et al. (2012)^d^ [18]	11,742	6.34 (0.03)	Low	**46.0**		NR		12.0
28	Campanini M et al. (2017)^d^ [67]	1596	7.55 (1.65)	Moderate	**48.7**		22.9		28.3
29	Alemán et al. (2016)^e^ [24]	1732	4.6 (3.3–6.0)^j^	Moderate	NR				
30	León-Muñoz et al. (2014)^a,d^ [46]	1815	MEDAS: 7.18 MDS: 4.93	Moderate Moderate	NR				
31	Rodríguez-Mireles S et al. (2018)^e^ [27]	2009: *n* = 4160 2015: *n* = 4143	2009: 5.20 (1.66) 2015: 5.17 (1.84)	2009: Moderate 2015: Moderate	NR				
32	Moreno-Agostino D et al. (2019)^d^ [68]	2397	8.55 (1.95)	Moderate	NR				
33	Garcıa-Arenzana et al. (2012)^a^ [38]	3564	5 (4–6)^j^	Moderate	NR				
10	Buckland et al. (2010)^b^ [30]	40,641	11 (2.4)	High	NR				
	**Italy**								
34	Dinu M et al. (2020)^i^ [69]	1820	12.18 (2.40)	Moderate	NR				
35	Ruggiero E et al. (2019)^c^ [17]	7430	29.6 (5.4)	Low	33.8		29.7		**36.5**
36	Barrea L et al. (2017)^d^ [70]	1013	7.1 (3.0)	Moderate	37.7		**39.3**		23
37	Limongi F et al. (2017) ^h^ [71]	4232	NR		31.9		26.1		**41.7**
38	Zappala G et al. (2019)^b^ [55]	1936	NR		**86.6**		NR		14.1
39	Marventano S et al. (2018)^b^ [56]	1937	NR		23.8		**61.9**		14.2
40	Bertoli et al. (2015)^d^ [72]	4388	7.0 (5.0–8.0)	Moderate	NR		NR		13.6
41	Bonaccio et al. (2012)^a,e^ [47]	13,262	MDS^a^: 4.44 (1.64) IMI^e^: 3.26 (1.71)	Moderate Moderate	NR				
10	Buckland et al. (2010)^b^ [30]	45,201	11 (2.3)	High	NR				
	**Multinational (Malta, Cyprus, Croatia, Lebanon, Israel and MD islands)**								
42	Cuschieri S et al. (2021)^i^ [73]	3947	7.19 (1.91)	Low	**40**		37.7		24.3
43	Kyprianidou et al. (2020)^c^ [62]	1123	15 (13–18)^j^	Low	32.6		**36.7**		30.5
44	Quarta S et al. (2021)^d^ [74]	2163	7.08 (1.96)	Moderate	20.7		**68.3**		11
45	Foscolou A et al. (2018)^c^ [60]	3131	32.5 (5.0)	Low	NR				
46	Cherfan M et al. (2018)^c^ [61]	2014	30.9 (4.6)	Low	NR				
47	Kolčić et al. (2016)^f^ [75]	2768	11 (8–13)^j^	Moderate	NR				
48	Naja et al. (2015)^a,b,c,e^ [32]	2048	LMD^g^:17.38 (3.40) MedDietScore^c^: 27.23 (4.65) IMI^e^: 3.56 (1.76) rMED^b^: 8.27 (2.49) MDS^a^: 4.18 (1.49)	Moderate Low Moderate Moderate Moderate	NR				
49	Zbeida et al. (2014)^a^ [48]	1786	NR		26.7		**62.1**		11.2

*NR* not reported

^a^Mediterranean Diet Scale (MDS) by Trichoupoulo et al. 0-9/0-8

^b^rMed by Buckland et al. 0-17/0-18

^c^MedDietScore by Panagiotakos et al. 0-55

^d^MEDAS by Schroder et al. 0-14

^e^Italian Mediterranean Index (IMI) by Agnoli et al. 0-10/0-11

^f^Mediterranean Diet Scoring System (MDSS) by Monteagudo et al: 0-24

^g^Lebanese Mediterranean Index (LMD) by Naja F et al. 9-27

^h^Mediterranean diet score (MDS) by Goulet J et al. 0-44

^i^Medi-Lite score by Sofi et al. 0-18

^j^Median (Inter-quartile range)

^k^The categories with the highest percentage are highlighted in bold, studies: 5, 13, 17, 35 presented a distribution in 4 categories for harmonization purposes we combined the second and third category

### Quality analysis of the included studies

The quality analysis of the included studies done as per the NCCMT scale [28] yielded the following results: Twenty-eight studies had a weak rating on the selection bias criterion evaluated regarding the representativeness of the sample toward the general population and regarding whether the study failed to report on the percentage of the population that agreed to participate in the study. Twenty-one studies scored moderate on selection bias and only one had a high-quality score. Concerning the validity and reliability of the data collection tools, 34 studies had a moderate score where the collection tools used were valid but their reliability was not documented, 15 studies had a weak score, and only two papers had a strong score. The last parameter of the quality analysis, related to withdrawals and drop-out rates, was applicable to 32 out of the 50 studies, 14 of which had a weak score, where drop-outs and withdrawals were either not reported or the percentage of participants who completed the study was higher than 60%. Twelve had a high score, with 80–100% of enrolled participants completing the study, and six had a moderate score, with 60–80% of participants completing the study. In total, 16 out of the 50 included studies had an overall weak quality score and 17 had a moderate score. More details about the quality assessment of the included studies are provided in supporting information 1 (S1).

### MD adherence

Of the 36 studies that reported mean or median MD adherence scores, 26 reported moderate adherence to MD, eight reported low adherence, one study [32] reported moderate adherence as per the MDS, rMed, IMI and LMD scores, and low adherence as per the MedDietScore, and one study [30] reported high adherence. The latter study was a multinational study including four European Mediterranean countries (France, Greece, Italy, and Spain), reporting high adherence among the Greek, Italian and Spanish populations and moderate adherence among French females. Studies that reported the distribution of the sample according to categories (e.g., low, moderate and high) also found low (*n* = 7) or moderate (*n* = 20) MD adherence as the most prevalent categories, and three studies found high adherence.

### MD adherence in subgroups

#### Sex groups

Twenty-one studies reported mean MD adherence for men and women separately [8, 16, 20, 24, 39, 41, 42, 44–46, 51, 55, 56, 59, 61, 62, 64, 69, 72–74, 76, 77]. Nine of these papers reported moderate adherence for both sex [16, 39, 41, 42, 44, 69, 72, 76, 77], while one paper reported high adherence [56] and three reported low adherence for both sex [61, 62, 73]. One paper reported high adherence in women and moderate adherence in men [51], while another reported low adherence in women and moderate adherence in men [59] and in contrast one reported low adherence in men and moderate adherence in women [74]. Out of the three studies that included only females, two [46, 64] reported high adherence and one [55] reported low adherence to the MD. Finally, one [20] study reported moderate adherence for men over the years (between 1985–1989 and 2005–2006), in contrast to a decrease in adherence for women from moderate during 1985–1989 to low during 2005–2006. More details about mean MD adherence and/or distribution per sex subgroups are provided in supporting information 2 (S2).

#### Age

Eight papers reported mean MD adherence scores per age group [20, 38, 56, 60, 62, 69, 73, 74]. The MD adherence scores were low for all age categories in two papers [62, 73] and moderate in three papers [20, 60, 69], while one paper that included females aged between 45–68 years only reported lower scores among the youngest women [38] and one paper reported low adherence among the age groups 18 to 44 and moderate for the participants aged more than 45 [74]. One study reported high adherence across age groups (18 + years old) [56]. Out of the nine studies that included a sample composed of the elderly aged 65 years and above, six reported moderate MD adherence [16, 39, 48, 57–59], two studies [8, 60] reported a low adherence level, and one study [71] found that the majority of the population (41.7%) were classified in the highest category of adherence. More details about mean MD adherence and/or distribution per age subgroups are provided in supporting information 3 (S3).

## Discussion

This systematic review provided an overview of MD adherence among the general adult population living in a Mediterranean country that traditionally follow a Mediterranean diet. The search strategy identified 50 articles that fulfilled the inclusion criteria, mostly reporting moderate MD adherence.

The majority of studies reported low to moderate MD adherence (35 studies). This seems to indicate that Mediterranean populations seem to be shifting away from the MD. Moreover, moderate adherence was also found to be the most prevalent adherence category in studies that provided data on distribution of the population across categories of MD adherence. This indicates that the moderate MD adherence score results from most people being in the moderate category rather than from one part of the population scoring very high and the other part of the population scoring very low. The only study reporting high MD adherence was a multinational prospective cohort (the EPIC cohort) by Buckland et al. conducted in nine European countries (four of them being Mediterranean countries: France, Spain, Italy, and Greece) [30]. This study reports on data collected between 1992 till 2000, which is less recent than most of the other papers included in this review. However, it is unclear whether this reflects an actual decrease in adherence, as Kyriacou et al. [78] highlighted the impact of the scoring system on the measured level of adherence: adherence as evaluated by the MDS of Trichopoulou et al. [12], the first scoring system available, seems to obtain higher values of MD adherence.

On another note, MD adherence scores are potentially being diluted in national studies due to the variability of the typical diet within each of the Mediterranean countries (e.g., northern vs southern regions). For instance, the population of southern Italy showed better MD adherence than inhabitants of northern Italian regions [17]. To understand the reasons underlying the levels of MD adherence, potential determinants of adherence such as age, place of residency, sex, and socio-economic and educational status should be further examined, calling for an investigation on the determinants of MD adherence across Mediterranean countries.

Concerning MD adherence in subgroups, in general, few sex differences were found in the 23 papers reporting on MD adherence for men and women separately. Most of the papers reported moderate to low adherence in both sexes. This finding is shared by the systematic review by Kyriacou et al. on MD adherence in Greek and Cypriot populations, reporting no difference between sex groups [78]. As an exception, in their paper published from the ATTICA cohort, Arvaniti et al. found that there were higher scores among women compared to men, mainly due to higher intakes of fruits and dairy products and lower intakes of red meat among women [23]. It is important to identify such potential sex differences in MD adherence to develop and implement sex-sensitive interventions to promote MD among both men and women. Such a tailoring of nutritional interventions by sex is reported to result in better adherence to a dietary pattern [79].

This systematic review also sheds some light on MD adherence in different age groups. Based on studies in this review, there does not seem to be a difference according to age: MD adherence is moderate in all age groups. This contrasts with previous hypotheses, as it is expected that older generations stick to the more traditional diets, whereas younger generations turn to more Western diets [80]. One study by Garcıa-Arenzana et al. [38] reported significantly lower scores among the youngest women, which joins the work of Veronese et al. [20], that reported a stronger decrease in adherence to MD among the younger population compared to the older population, due to special dietary modifications related to age, as a consequence of the increase in nutrition-related disorders (e.g., cardiovascular diseases, diabetes). In contrast, according to Foscolou et al. [60], there was a decrease in MD adherence among the older population over the years (between 2005 and 2017) and this was attributed to various social determinants: the place of residency, level of education, and lifestyle factors [60]. This systematic review cannot draw definite conclusions on the level of adherence among different age groups. More insight is needed in MD adherence scores across ages and determinants for shifting away from the MD.

The vast majority of studies in this systematic review pertained to France, Spain, Greece, and Italy. We found a gap in this area of research among the African Mediterranean countries (Egypt, Libya, Tunisia, Algeria, and Morocco) as well as the Middle Eastern Mediterranean countries (Syria, Lebanon, Israel, and Palestinian territory). This could be due to one of the inclusion criteria (> 1000 participants), but there were also few studies from non-European countries in the excluded studies with small sample sizes.

The quality assessment showed weak to moderate scores on selection bias, validity and reliability of measurement instruments, and withdrawal and drop-out criteria from the NCCMT scale [28] for assessment of quantitative studies for most of the included studies. This indicates the need for more high-quality studies among Mediterranean countries, especially in terms of representativeness of the general population, the use of validated and reliable measurement tools, and the MD adherence score, to get a more accurate picture of the adherence to MD among Mediterranean populations.

This systematic review has several strengths: it followed the PRISMA guidelines and was registered in the Prospero database (CRD42020189337). Multiple databases were searched: PubMed, Web of Science and PsycINFO, to collect a broad range of articles from different research fields. In addition, the screening, data extraction, and quality assessment were performed by independent researchers, and the tool used to assess the quality of the included studies was a validated instrument [28]. Nevertheless, there are some limitations to our systematic literature review. First of all, the search was restricted to studies published in the English language. Moreover, studies including less than 1000 participants were excluded, which in practice prevented us from capturing the MD adherence status from various smaller studies from several North African and Middle Eastern Mediterranean countries. Further, a variety of scores was used to evaluate Mediterranean diet adherence and the categorization was also variable, and there was no clear definition of cut-off points to classify adherence to MD: some studies used tertiles, others used quartiles, and some set their own cut-offs to classify the adherence as low, moderate or high. The variability of the indexes in terms of range, and food items included in the calculation of the score have made it hard to trace and/or to compare the adherence level between countries or within the same country. This is well described in a systematic review by Zaragoza-Marti et al., who found 28 different MD adherence scores that had variable psychometric properties [81]. This led us to create a system of classification where MD adherence scores were categorized as low, medium or high. However, this system of classification might have affected the interpretation of means, since various studies reported mean MD scores at the borderline of the different categories, which might have led us to label a study as reporting low, moderate or high MD adherence with only a decimal value difference in the mean score.

## Conclusion

This is the first paper that systematically reviewed the scientific literature available on MD adherence among adults living in Mediterranean countries specifically. Mediterranean populations have been showing low to moderate adherence to MD in the past 10 years. Therefore, there is an urgent need to improve adherence to MD by younger and older adults, and for men and women, even in the countries of its origin. This requires appropriate health promotion and nutritional policies and interventions. Health promotion efforts to improve adherence to MD can have large effects on a broad range of health outcomes of inhabitants [2, 6, 7]

The large variety of indexes used to measure MD adherence, in addition to variability in the classification systems, does not enable us to compare and trace MD adherence between and within countries, as well as across time. We therefore recommend establishing a common system of classification for the MD adherence indexes. We also advocate the implementation of large-scale studies of MD adherence in African Mediterranean and Middle Eastern Mediterranean countries, as they were underrepresented in the current review. In addition, to be able to improve MD adherence, it is important to study determinants of adherence to the MD in these countries to help in designing appropriate local and national policies and interventions to promote adherence to the MD. Therefore, we recommend a systematic review of the literature on determinants of adherence to the MD in Mediterranean countries. This would enable health promoters to intervene and improve the adherence level to this healthy dietary pattern, based on identified determinants.

## Supplementary Information

Below is the link to the electronic supplementary material.

Supplementary file1 (PDF 111 KB)

Supplementary file2 (DOCX 55 KB)
