# Supplementary material for: Adherence to the Mediterranean diet among adults in Mediterranean countries: a systematic literature review
Source: Eur J Nutr. 2022 Apr 22;61(7):3327–44. doi: 10.1007/s00394-022-02885-0 (PMC9026058; doi:10.1007/s00394-022-02885-0)
Supplement: Supplementary file 2 — Supplementary file2 (DOCX 55 KB) [file 394_2022_2885_MOESM2_ESM.docx]

**Title: Adherence to the Mediterranean diet among adults in Mediterranean countries: a systematic literature review**

**Journal: European Journal of Nutrition**

**Cecile A. Obeid, Jessica S. Gubbels, Doris Jaalouk, Stef P.J. Kremers, Anke Oenema**

**Cecile A. Obeid***

- NUTRIM School of Nutrition and Translational research in metabolism, Department of Health Promotion, Maastricht University Medical Centre, PO Box 616, 6200 MD Maastricht, the Netherlands
- Faculty of Nursing and Health Sciences, Notre Dame University, Zouk Mosbeh, Lebanon P.O. Box: 72, Zouk Mikael Lebanon
- ORCID ID: 0000-0001-9538-282X

**Jessica S. Gubbels**

- NUTRIM School of Nutrition and Translational research in metabolism, Department of Health Promotion, Maastricht University Medical Centre, PO Box 616, 6200 MD Maastricht, the Netherlands

**Doris Jaalouk**

- College of Arts & Sciences, American University of Iraq Baghdad (AUIB), Baghdad Airport Road, Baghdad, Iraq

**Stef P.J. Kremers**

- NUTRIM School of Nutrition and Translational research in metabolism, Department of Health Promotion, Maastricht University Medical Centre, PO Box 616, 6200 MD Maastricht, the Netherlands

**Anke Oenema**

- NUTRIM School of Nutrition and Translational research in metabolism, Department of Health Promotion, Maastricht University Medical Centre, PO Box 616, 6200 MD Maastricht, the Netherlands

* Correspondence: Cecile A. Obeid, Faculty of Nursing and Health Sciences, Notre Dame University, Zouk Mosbeh, Lebanon P.O. Box: 72, Zouk Mikael Lebanon, Email: [cobeid@ndu.edu.lb](mailto:cobeid@ndu.edu.lb)

Telephone: +961 3 877121

Mailing address: ESLA 5116, first floor, Mar Takla - Saed Freiha street, Hazmieh, Lebanon, Postal code: 1107-2090.

**Supporting Information**

The following Supporting Information is available through the online version of this article at the publisher’s website.

*Appendix S1:* Table of quality analysis of included studies

Appendix S2: Table of mean distribution by sex

*Appendix S3:* Table of mean distribution by age group

*Appendix S4:* Search strategy per database

Supporting information 2 (S2): Table of mean distribution by sex

|  | | | classification of mean | | | **Distribution of population (%) by categories of MD adherence** | | | | | | | |
| --- | --- | --- | --- | --- | --- | --- | --- | --- | --- | --- | --- | --- | --- |
| Reference | mean (SD) | | low | moderate | high | category 1 | | category 2 | | category 3 | | category 4 | |
|  | *Men* | *Women* |  |  |  | *Men* | *Women* | *Men* | *Women* | *Men* | *Women* | *Men* | *Women* |
| Couto E et al. (2011) ^(a)^ |  |  |  | Across genders in different countries |  | 0-3 total med countries: 29.5 Greece: 29.9 Spain: 29.3 Italy: 29.5 | 0-3: total med countries: 32.2 Greece: 35. Spain: 34.2 Italy: 34.1 France: 30.1 | 4: total med countries: 21.8, Greece: 21.4, Spain: 22 Italy: 21.8 | 4: total med countries: 29.3 Greece: 22.1. Spain: 22.8 Italy: 21.1 France: 22.6 | 5: total med countries: 21.3, Greece: 21 Spain: 21.6 Italy: 21.3 | 5: total med countries: 21.1 Greece: 20.4. Spain: 21.1 Italy: 20 France: 21.7 | 6-9: total med countries: 27.3, Greece: 27.7, Spain: 27 Italy: 27.5 | 6-9: total med countries: 24.4 Greece: 22.5. Spain: 21.9 Italy: 24.9 France: 25.6 |
| Feart C et al. (2011) ^(a)^ | 4.5 (1.7) | 4.2 (1.6) |  | men and woman |  | 0-3: 25.6 | 0-3: 32.6 | 4-5: 44.6 | 4-5: 43 |  |  | 6-8: 29.8 | 6-8: 24.3 |
| Feart C et al. (2012) ^(a)^ | 4.54 (1.72) | 4.25 (1.63) |  | men and woman |  |  | 0-3: 66.1 |  | 4-5: 62.9 |  |  |  | 6-9: 55.4 |
| Martı´nez-Gonza´lez et al. (2012) ^(a)^ |  |  |  | men and woman |  | 0-2: 17.6 | 0-2: 19.2 | 3-5: 56.0 | 3-5: 58.0 |  |  | 6-9: 26.4 | 6-9: 22.8 |
| Veronese N et al. (2020) ^(b)^ | 1985-89: age group 30 - 49: 31.5 age group 50 - 69: 32.1 2005-06: age group 30 - 49: 29.93 age group 50 - 69: 30.9 | 1985-89: age group 30 - 49: 32.23. age group 50 - 69: women: 32.32. 2005-2006: age group 30 - 49: 28.21 age group 50 - 69: 29.11 | men across years and age groups and woman across age groups in 2005-06 | year 1985-89: woman |  |  |  |  |  |  |  |  |  |
| Gutiérrez-Carrasquilla L et al. (2019) ^(c)^ |  |  |  |  | woman |  | ≤6: 41.0 |  | 7 - 10: 57.9 |  |  |  | 11: 60.7 |
| Zappala G et al. (2019) ^(d)^ |  |  | woman |  |  | 86.2 | 85.7 |  |  |  |  | 13.8 | 14.3 |
| Maraki M et al. (2019) ^(b)^ | 34.3 (4.3) | 32.5 (4.7) | woman | men |  |  |  |  |  |  |  |  |  |
| Olza J et al. (2019) ^(a)^ |  |  |  |  |  | MDS < 4: 37.9 MDS-Mod < 4: 32.1 | MDS < 4: 51.1 MDS-Mod < 4: 34.2 | MDS-Mod 4-5: 42.3 | MDS-Mod 4-5: 42.4 |  |  | MDS ≥4: 62.1 MDS-Mod >6: 25.6 | MDS ≥4: 48.9 MDS-Mod >6: 23.3 |
| Adjibade M et al. (2018) ^(d)^ |  |  |  | men | woman | 1–7: 28.3 | 0–7: 32.1 | 8–10: 39.5 | 8–9: 28.2 |  |  | 11–17: 32.2 | 10–16: 39.7 |
| Marventano S et al. (2018) ^(d)^ | 11.9 (2.4) | 12.0 (2.3) |  |  | men and woman |  |  |  |  |  |  |  |  |
| Cherfan M et al. (2018) ^(b)^ | 30.5 (4.7) | 31.4 (4.4) | men and woman |  |  |  |  |  |  |  |  |  |  |
| Mateo-Gallego R et al. (2017) ^(a)^ | 4.19 (1.70) | 4.24 (1.72) |  | men and woman |  |  |  |  |  |  |  |  |  |
| Alemán et al. (2016) ^(a)^ |  | no gender differences |  |  |  |  |  |  |  |  |  |  |  |
| Lelong et al. (2016) ^(a)^ | 4.4 (1.6) P=0.004 | 4.2 (1.6) P=0.004 |  | men and woman |  |  |  |  |  |  |  |  |  |
| Bertoli et al. (2015) ^(c)^ | 7.0 (6.0-8.0) | 7.0 (5.0-8.0) |  | men and woman |  |  |  |  |  |  |  |  |  |
| León-Muñoz et al. (2014) ^(a,c)^ |  |  |  |  | woman |  | MEDAS: 56.9 MDS: 58.7 |  |  |  |  |  | MEDAS: 61.0 MDS: 62.2 |
| Lassale et al. (2012) ^(a,d)^ | MDS: 4.6 (1.6) rMED: 9.2 (2.8) | MDS: 4.4 (1.6)  rMED: 8.7 (2.8) |  | men and woman |  |  |  |  |  |  |  |  |  |
| Dinu M et al. (2020) ^(e)^ | 11.85 ± 2.37 | 12.39 ± 2.39 |  | Men and woman |  |  |  |  |  |  |  |  |  |
| Kyprianidou et al. (2020)^(b)^ | 16 (13-19)^(f)^ | 15 (12-18) ^(f)^ | Men and woman |  |  | <13: 26.8 | <13: 37.3 | 13-17: 38.0 | 13-17: 35.9 |  |  | >17: 35.2 | >17: 26.8 |
| Cuschieri S et al. (2021)^(e)^ | 7.45 (1.96) | 6.93 (1.81) | Men and woman |  |  | ≤6: 58.2 | ≤6: 41.8 | 7–8: 49.5 | 7-8: 50.5 |  |  | 9+: 40.8 | 9+: 59.2 |
| Mantzorou et al. (2021)^b^ |  |  |  |  |  | ≤25: 15.0 | ≤25: 11.3 | 26-28: 13.4 | 26-28: 12.4 | 29-31: 11.9 | 29-31: 13.0 | ≥32: 7.9 | ≥32: 15.1 |
| Quarta S et al. (2021)^c^ | 6.81 ± 2.01 | 7.22 ± 1.92 | Men | Women |  | ≤5: 24.1 | ≤5: 19 | 6-9: 67.3 | 6-9: 68.8 |  |  | ≥10: 8.6 | ≥10: 12.2 |

^(a)^ Mediterranean diet scale (MDS) by Trichoupoulo et al. 0-9/0-8, ^(b)^ MedDietScore by Panagiotakos et al. 0-55, ^(c)^ MEDAS by Schroder et al. 0-14, ^(d)^ rMed by Buckland et al. 0-17/0-18, ^(e)^ Medi-Lite by Sofi et al. 0-18, ^(f)^ median (IQR)

Supporting information 3 (S3): Table of mean distribution by age groups

|  |  | Classification of mean | | |
| --- | --- | --- | --- | --- |
| Reference | Mean (SD) by age group | *Low* | *Moderate* | *High* |
| Veronese N et al. (2020) ^(b)^ | Age group 30 - 49: 1985-89: 31.82 (4.18) 2005-2006: 29.20 (4.48) age group 50-69: 1985-89: 32.20 (4.09) 2005-2006: 30.15 (4.27) |  | across ages |  |
| Marventano S et al. (2018) ^(c)^ | <30: 11.5 (2.4) 30–39: 11.6 (2.5) 40–49: 12.1 (2.4) 50–59: 12.1 (2.4) 60–69: 12.6 (1.9) ≥70: 12.1 (2.4) |  |  | across ages |
| Foscoloua A et al. (2018) ^(b)^ | 65–69 y (n = 1171) 33 (4.8) 70–74 y (n = 680) 32.4 (5.0) 75–79 y (n = 630) 32.4 (4.9) 80–84 y (n = 390) 31.9 (5.4) 85–89 y (n = 173) 32.5 (5.2) ≥90 y (n = 76) 32.1 (5.3) | 70 and above | 65 -69 |  |
| Garcı´a-Arenzana et al. (2012) ^(a)^ | In the case of the aMED index, lower scores were observed among the youngest women (P for trend < 0.001) |  |  |  |
| Dinu M et al. (2021) ^(d)^ | 18–30: 11.97 ± 2.40  31–45: 12.37 ± 2.24  46–60: 12.33 ± 2.48  >60: 12.37 ± 2.48 |  | across ages |  |
| Kyprianidou et al. (2020)^(b)^ | 18-24: 16(13-18)^(f)^  25-44: 15(13-18)  45-64: 15(12-18)  65+: 16(13-18) | Across ages |  |  |
| Cuschieri S et al. (2021)^(d)^ | <30: 7.11 (1.83)  30–44: 6.89 (1.87)  44–65: 7.36 (1.92)  >65: 7.47 (1.97) | Across ages |  |  |
| Quarta S et al. (2021)^(e)^ | 18-24: 6.53 ± 1.88  25-44: 6.91 ± 1.92  45-64: 7.56 ± 1.96  ≥ 65: 7.89 ± 1.82 | 18-24  25-44 | 45-64  ≥ 65 |  |

^(a)^ Mediterranean diet scale (MDS) by Trichoupoulo et al. 0-9/0-8, ^(b)^ MedDietScore by Panagiotakos et al. 0-55, ^(c)^ rMed by Buckland et al. 0-17/0-18, ^(d)^ Medi-Lite by Sofi et al. 0-18, median (IQR), ^(e)^ MEDAS by Schroder et al. 0-14, ^(f)^ Median

Supporting information 4 (S4): Complete search strategy

| Database | Search strategy* |
| --- | --- |
| Medline/ PubMed  24/01/2022^a^ | ((Mediterranean diet[MeSH^b^ Terms] OR "Mediterranean diet"[Title/Abstract] OR "Mediterranean dietary pattern"[Title/Abstract])) AND((adherence[Title/Abstract] OR compliance[Title/Abstract] OR observance[Title/Abstract] OR prevalence[Title/Abstract])) |
| PsychINFO^f^  24/01/2022 | (TI^c^ Mediterranean diet OR AB^d^ Mediterranean diet OR MA Mediterranean diet OR TI Mediterranean dietary pattern OR AB Mediterranean dietary pattern)  **AND** (TI adherence OR AB adherence OR TI compliance OR AB compliance OR TI observance OR AB observance OR TI prevalence OR AB prevalence) |
| Web of Science  24/01/2022 | (TS=Mediterranean diet OR "Mediterranean dietary pattern") AND  ((TI=(adherence OR observance OR compliance OR prevalence) OR AB=(adherence OR observance OR compliance OR prevalence )) |

^(a)24-01-2022= Date the search strategy was carried out; (b)MeSH = Medical Subject Headings; (c)TI= Title; (d)AB= Abstract; (e)TS = Topic; (f)PsycINFO = Psychology Information.* applied filters: English language, publication date from 2000/01/01, age groups: adulthood (18 years and older).^

Supporting information 1 (S1): Table of quality analysis of included studies

|  | **Authors** | **Year** | **A Q1 Are the individuals selected to participate in the study likely to be representative of the target population? ^(a)^** | **A Q2 What percentage of selected individuals agreed to participate? ^(b)^** | **A Section Selection bias Rating ^(c)^** | **E Q1 Were the data collection tools shown to be valid?^(d)^** | **E Q2 Were data collection tools shown to be reliable^? (e)^** | **E Section Data collection methods Rating ^(f)^** | **F Q1 Were withdrawals and drop-outs reported in terms of numbers and/or reasons per group?^(g)^** | **F Q2 Indicate the percentage of participants completing the study (if the percentage differs by groups, record the lowest) ^(h)^** | **F Section Withdrawals and drop-outs Rating ^(i)^** | **Overall rating** |
| --- | --- | --- | --- | --- | --- | --- | --- | --- | --- | --- | --- | --- |
| 1 | Féart, C. et al. | 2011 | Somewhat likely | Can't tell | Moderate | Can't tell | Can't tell | Weak | Yes | Less than 60% | Weak | Weak |
| 2 | Féart, C. et al. | 2012 | Somewhat likely | Can't tell | Moderate | Can't tell | Can't tell | Weak | Yes | Less than 60% | Weak | Weak |
| 3 | Kesse-Guyot, E. et al. | 2013 | Not likely | Less than 60% agreement | Weak | Yes | Can't tell | Moderate | Yes | Less than 60% | Weak | Weak |
| 4 | Barré et al | 2017 | Not likely | Can't tell | Weak | Yes | Can't tell | Moderate | Yes | 60-79% | Moderate | Moderate |
| 5 | Trébuchet, A. et al. | 2019 | Not likely | Can't tell | Weak | Yes | Can't tell | Moderate | Yes | 80-100% | Strong | Moderate |
| 6 | Adjibade, M. et al. | 2018 | Not likely | Less than 60% agreement | Weak | Yes | Can't tell | Moderate | Yes | Less than 60% | Weak | Weak |
| 7 | Lavalette, C. et al. | 2018 | Not likely | Can't tell | Weak | Yes | Can't tell | Moderate | Yes | Less than 60% | Weak | Weak |
| 8 | Lelong et al. | 2016 | Not likely | Can't tell | Weak | Yes | Can't tell | Moderate | No | Can't tell | Weak | Weak |
| 9 | Lassale et al. | 2012 | Not likely | Can't tell | Weak | Can't tell | Can't tell | Weak | Yes | Can't tell | Moderate | Weak |
| 10 | Buckland, G. et al. | 2010 | Not likely | Can't tell | Weak | Yes | Can't tell | Moderate | Yes | 80-100% | Strong | Moderate |
| 11 | Mamalaki et al | 2019 | Not likely | Can't tell | Weak | Yes | Can't tell | Moderate | No | Can't tell | Weak | Weak |
| 12 | Mamalaki et al | 2018 | Somewhat likely | Can't tell | Moderate | Yes | Can't tell | Moderate | No | Can't tell | Weak | Moderate |
| 13 | Maraki et al. | 2018 | Somewhat likely | Can't tell | Moderate | Yes | Can't tell | Moderate | No |  | Weak | Moderate |
| 14 | Panagiotakis, D. B. et al. | 2015 | Can't tell | 60-79% agreement | Weak | Yes | Can't tell | Moderate | Yes | 80-100% | Strong | Moderate |
| 15 | Gutiérrez-Carrasquilla et al. | 2019 | Not likely | Can't tell | Weak | Yes | Can't tell | Moderate | Can't tell |  | Weak | Weak |
| 16 | Navarrete-Munoz et al. | 2018 | Not likely | Can't tell | Weak | Yes | Can't tell | Moderate | Can't tell |  | Weak | Weak |
| 17 | Galilea-Zabalza et al | 2018 | Not likely | Can't tell | Weak | Can't tell | Can't tell | Weak | Yes | 80-100% | Strong | Weak |
| 18 | Cornejo del Río et al | 2017 | Somewhat likely | 60-79% agreement | Moderate | Yes | Can't tell | Moderate | Can't tell |  | Weak | Moderate |
| 19 | Ferreira‑Pêgo et al. | 2017 | Somewhat likely | Can’t tell | Moderate | Yes | Can’t tell | Moderate | Not applicable (i.e. one time surveys) |  | Not applicable | NA |
| 20 | Domínguez, L.J. et al. | 2013 | Not likely | Can't tell | Weak | Yes | Can't tell | Moderate | Yes | 80-100% | Strong | Moderate |
| 21 | Mateo-Gallega, R et al. | 2017 | Not likely | Less than 60% agreement | Weak | Yes | Can't tell | Moderate | Not applicable (i.e. one time surveys) |  | Not applicable | NA |
| 22 | Olza et al | 2019 | Somewhat likely | Can't tell | Moderate | Can't tell | Can't tell | Weak | Not applicable (i.e. one time surveys) |  | Not applicable | NA |
| 23 | Sayón-Orea et al. | 2014- | Not likely | Can't tell | Weak | Yes | Can't tell | Moderate | Not applicable (i.e. one time surveys) |  | Not applicable | NA |
| 24 | León-Munoz, L.M. et al. | 2012 | Very likely | Less than 60% agreement | Weak | Can't tell | Can't tell | Weak | Not applicable (i.e. one time surveys) | 80-100% | Strong | Weak |
| 25 | Campanini et al | 2017- | Not likely | Can't tell | Weak | Yes | Can't tell | Moderate | No | Can't tell | Not applicable | NA |
| 26 | Abellán Alemán et al. | 2016 | Very likely | Can't tell | Moderate | No | Can't tell | Weak | Not applicable (i.e. one time surveys) |  | Not applicable | NA |
| 27 | León-Munoz, L.M. et al. | 2014 | Very likely | Can't tell | Moderate | Yes | Can't tell | Moderate | Yes | 60-79% | Moderate | Moderate |
| 28 | Rodríguez-Mireles et al. | 2018 | Somewhat likely | Can't tell | Moderate | Can't tell | Can't tell | Weak | Not applicable (i.e. one time surveys) |  | Not applicable | NA |
| 29 | Moreno-Agostino et al. | 2019- | Very likely | Can't tell | Moderate | Yes | Can't tell | Moderate | Yes | 60-79% | Moderate | Moderate |
| 30 | García-Arenzana et al. | 4-Jul | Somwhat likely | 60-79% agreement | Moderate | Yes | Can't tell | Moderate | Not applicable (i.e. one time surveys) |  | Not applicable | NA |
| 31 | Ruggiero, E. et al. | 2018 | Not likely | Can't tell | Weak | Can't tell | Can't tell | Weak | Yes | 80-100% | Strong | Weak |
| 32 | Barrea et al | 2017 | Not likely | Can't tell | Weak | Yes | Can't tell | Moderate | Yes | 80-100% | Strong | Moderate |
| 33 | Limongi et al. | 2017 | Very likely | 5 Can't tell | Moderate | Can't tell | Can't tell | Weak | Yes | 60-79% | Moderate | Moderate |
| 34 | Zappalá et al. | 2019 | Somewhat likely | Can't tell | Moderate | Yes | Yes | Strong | Not applicable (i.e. one time surveys) |  | Not applicable | NA |
| 35 | Marventano et al. | 2018 | Somewhat likely | Can't tell | Moderate | Yes | Yes | Strong | Not applicable (i.e. one time surveys) |  | Not applicable | NA |
| 36 | Bertoli, S et al. | 2015 | Can't tell | Can't tell | Weak | Yes | Can't tell | Moderate | Not applicable (i.e. one time surveys) |  | Not applicable | NA |
| 37 | Bonaccio, M. et al. | 2012 | Very likely | Can't tell | Moderate | Yes | No | Moderate | Yes | 60-79% | Moderate | Moderate |
| 38 | Veronese, N. et al. | 2020 | Very likely | 60-79% agreement | Moderate | Yes | Can't tell | Moderate | Yes | 80-100% | Strong | Moderate |
| 39 | Foscolou, A et al. | 2018 | Not likely | Can't tell | Weak | Yes | Can't tell | Moderate | Not applicable (i.e. one time surveys) |  | Not applicable | NA |
| 40 | Cherfan et al. | 2018 | Very likely | Can't tell | Moderate | Can't tell | Can't tell | Weak | Yes | 80-100% | Strong | Moderate |
| 41 | Kolcic, I. et al. | 2016 | Not likely | Can't tell | Weak | Can't tell | Can't tell | Weak | Not applicable (i.e. one time surveys) |  | Not applicable | Weak |
| 42 | Naja et al. | 2015 | Very likely | 80-100% agreement | Strong | No | Can't tell | Weak | Not applicable (i.e. one time surveys) |  | Not applicable | NA |
| 43 | Zbeida et al. | 2014 | Somewhat likely | Can't tell | Moderate | Can't tell | Can't tell | Weak | Not applicable (i.e. one time surveys) |  | Not applicable | NA |
| 44 | Koustonida et al. | 2021 | Somewhat likely | Can’t tell | Moderate | Yes | Can’t tell | Moderate | Yes | 80-100% | Strong | Moderate |
| 45 | Mantzorou et al. | 2021 | Somewhat likely | Can’t tell | Moderate | Yes | Can’t tell | Moderate | No | Can’t tell | Weak | Moderate |
| 46 | Zazpe I et al. | 2021 | Not likely | Can’t tell | Weak | Yes | Can’t tell | Moderate | No | Can’t tell | Weak | Weak |
| 47 | Dinu M et al. | 2021 | Not likely | Not applicable | Weak | Yes | Can’t tell | Moderate | Not applicable |  | Not applicable | NA |
| 48 | Cuschieri S et al. | 2021 | Very likely | Less than 60% agreement | Weak | Can’t tell | Can’t tell | Weak | Not applicable (i.e. one time surveys) |  | Not applicable | Weak |
| 49 | Kyprianidou et al. | 2020 | Not likely | 80-100% agreement | Weak | Yes | Can’t tell | Moderate | Not applicable (i.e. one time surveys) |  | Not applicable | NA |
| 50 | Quarta S et al. | 2021 | Can’t tell | Can’t tell | Weak | Yes | Can’t tell | Moderate | Not applicable (i.e. one time surveys) |  | Not applicable | NA |

1. ^Very likely, somewhat likely, not likely, can’t tell^
2. ^80-100% agreement, 60-79% agreement, less than 60% agreement, not applicable, can’t tell^
3. ^Strong, Moderate, Weak^
4. ^Yes, no, can't tell^
5. ^Yes, no, can't tell, not applicable (i.e. one time surveys)^
6. ^Strong (no weak ratings), Moderate (one weak rating), Weak (two or more weak ratings)^
7. ^Yes, no, can't tell, not applicable (i.e. one time surveys)^
8. ^80-100%, 60-79%, less than 60%, can't tell, not applicable (i.e. retrospective study)^
9. ^Strong, Moderate, weak, not applicable^
